# Meta2DB: curated shotgun metagenomic feature sets and metadata for health state prediction

**DOI:** 10.1093/bioinformatics/btag422

**Published:** 2026-07-01

**Authors:** Car Reen Kok, Nisha J Mulakken, James B Thissen, Jose Manuel Martí, Ryan Lee, Jacob B Trainer, Andre R Goncalves, Hiranmayi Ranganathan, Aram Avila-Herrera, Crystal J Jaing, Nicholas A Be

**Affiliations:** Lawrence Livermore National Laboratory, Livermore, CA, United States; Lawrence Livermore National Laboratory, Livermore, CA, United States; Lawrence Livermore National Laboratory, Livermore, CA, United States; Lawrence Livermore National Laboratory, Livermore, CA, United States; University of California Merced, Merced, CA, United States; Lawrence Livermore National Laboratory, Livermore, CA, United States; Lawrence Livermore National Laboratory, Livermore, CA, United States; Lawrence Livermore National Laboratory, Livermore, CA, United States; Lawrence Livermore National Laboratory, Livermore, CA, United States; Lawrence Livermore National Laboratory, Livermore, CA, United States; Lawrence Livermore National Laboratory, Livermore, CA, United States

## Abstract

**Summary:**

Meta2DB is a curated metagenomic and metadata database that provides structurally consistent microbiome taxonomy feature count tables for 13 897 samples across 84 studies, 23 disease states, and 34 geographical locations. All samples were uniformly processed using a streamlined metagenomic classification pipeline that employs a unique and comprehensive reference database indexed to contain all sequences across all kingdoms of life that were present in the NCBI Nucleotide (nt) database retrieved on 4 January 2023. This pipeline leverages high-performance computing (HPC) resources at Lawrence Livermore National Laboratory and was used to process 50TB of publicly available raw metagenomic sequence data. Extensive metadata curation was carried out through a combination of manual curation and automated parsing, producing a consistent inter-study metadata table specifically structured to facilitate training of ML models for prediction of human health.

**Availability:**

Data is available at https://gdo-meta2db.llnl.gov/ and https://zenodo.org/records/17315984.

## 1 Introduction

The human microbiome represents a target with tremendous potential for diagnosing and predicting human health. Microbiome features have been indicated as prognostic factors for a range of disease states, including cancer, gut health disruption, neurodegenerative disease, environmental exposure, and mental health. Development of models that leverage microbiome features for prediction of human health states could facilitate tools with clinically actionable utility and identify microbiome-centric variables for therapeutic intervention.

Machine learning (ML), and specifically deep learning (DL), approaches have demonstrated potential in exploring and classifying microbiomes in the context of human health (Namkung 2020, Wang *et al*. 2021). Convolutional neural networks (CNNs) have demonstrated the capacity to predict various modes of disease outcomes such as disease severity in ulcerative colitis, cirrhosis and inflammatory bowel disease using microbiome-derived data (Lo and Marculescu 2019, Sharma *et al*. 2020, Fung *et al*. 2023).

Microbiome data employed for predictive modeling are often derived from metagenomic sequence data generated from the specimens of interest. Such profiling can involve amplicon sequencing, where a specific gene or region such as the bacterial 16S rRNA sequence, is amplified and assigned to microbial taxonomy. Alternatively, shotgun metagenomic sequencing can be performed, whereby all gDNA within an extracted specimen are sequenced and the resultant sequence reads are aligned to references via a metagenomic classification software. The output from such analyses is a feature table, where a taxonomic identifier for a given taxonomic rank (e.g., genus, species) is assigned a numeric value (number of classified sequences), which can then be transformed as desired and used as input for the ML platform of choice.

The high-dimensional nature of microbiome data derived from many features (p) alongside small sample sizes (n) often result in large p and small n datasets. This presents a critical challenge in ML modeling, resulting in overfitting and limiting generalizability to new or unseen data. One strategy for sample size expansion is to integrate publicly available datasets across different studies. While health-relevant microbiome datasets are becoming increasingly available, dataset integration for use in a cohesive model training approach is difficult as 1) microbiome feature tables are generated by distinct metagenomic classification platforms that impart systematic biases, 2) independent studies implement distinct quality and sequence filtering steps, and 3) metadata are inconsistently and sparsely represented.

One avenue for addressing this need is to perform a meta-acquisition of the raw sequencing data from multiple independent studies, and to process all such datasets through a unified metagenomic classification pipeline alongside harmonized metadata. Several resources have adopted this strategy, including the Human Microbiome Compendium (Abdill *et al*. 2025), the curatedMetagenomicData data package (Pasolli *et al.* 2017), the Metalog database (Kuhn *et al*. 2026) and the Human Long-Read Metagenomics Database (HLRMDB) (Zhai *et al*. 2026). These platforms provide consistently structured microbiome profiles across multiple studies using either 16S rRNA amplicon sequencing or shotgun metagenomic sequencing. The Human Microbiome Compendium pipeline incorporates the DADA2 algorithm (Callahan *et al*. 2016) to infer Amplicon Sequence Variants for taxonomic identification from 16S data. In contrast, curatedMetagenomicData uses MetaPhlAn3 (Beghini *et al*. 2021), which employs a marker library reference for profiling shotgun metagenomes, enabling higher taxonomic resolution while maintaining computationally efficiency relative to whole genome indexes. However, this strategy is inherently limited to the taxa represented in the marker database. Metalog extends these efforts by providing a curated repository of manually annotated metadata linked to MetaPhlAn4 and mOTUs-based metagenome profiles across diverse host-associated and environmental datasets. Meanwhile, HLRMDB integrates long-read, hybrid, and assembly-based approaches to generate strain-resolved profiles with accompanying functional annotations. Together, these resources underscores the value of large-scale, standardized curation of metagenomic data and associated metadata, enabling more robust and comparable meta-analyses.

**Figure 1:**
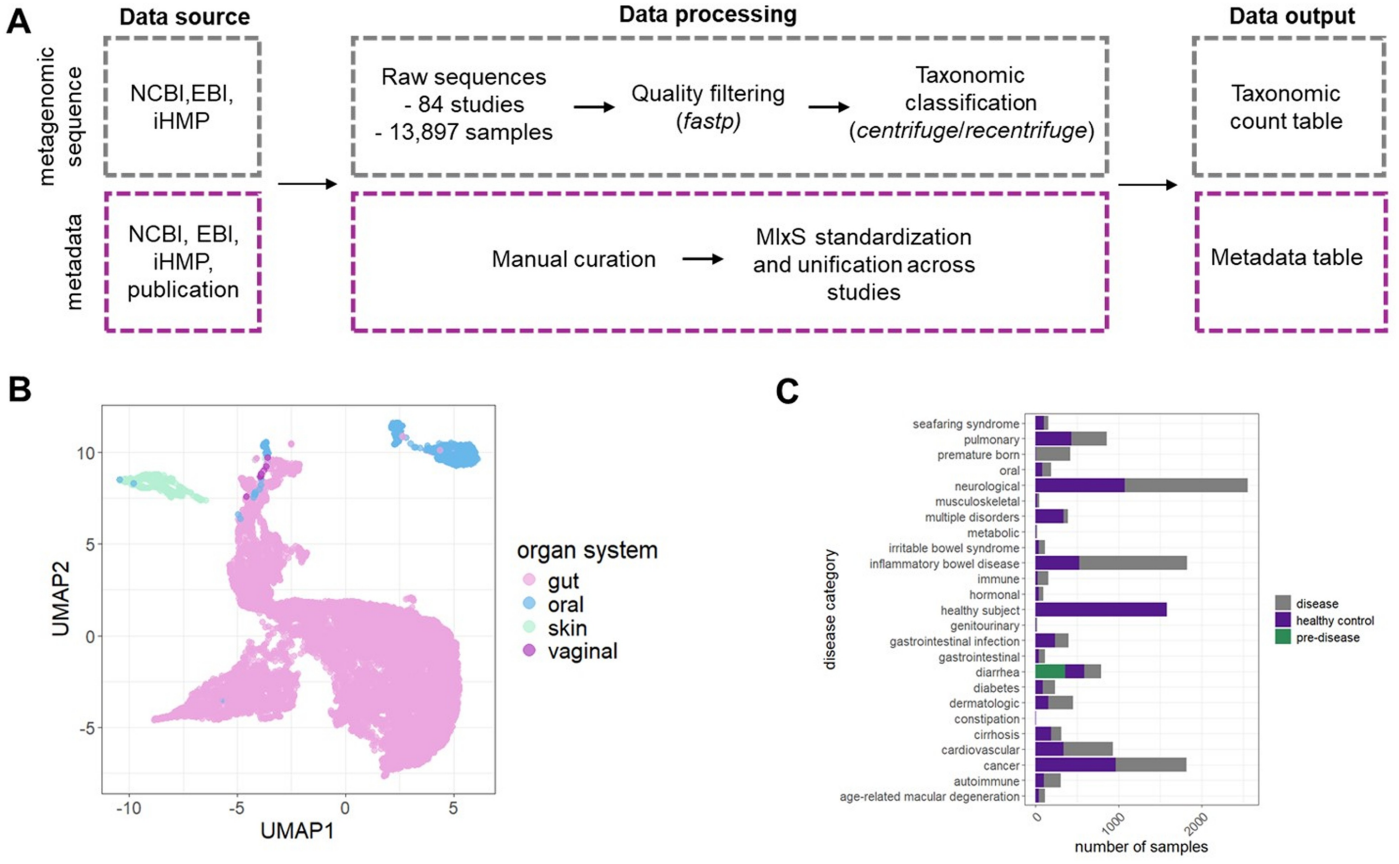
The Meta2DB pipeline enables the systematic curation of shotgun metagenomic data, producing labelled metagenomic profiles that can be directly used to train machine learning models. (A) Data intake, processing and integration of publicly available shotgun metagenomic sequences and associated metadata. (B) UMAP plot of 13,897 samples based on Bray-Curtis distances in relative abundance space, illustrating the distribution of samples and organ systems (gut, oral, skin, vaginal). Samples are colored according to different organ systems. As batch correction was not applied, clustering patterns may reflect both biological and technical variation. (C) Number of samples per disease category, colored by health and disease state labels.

Our approach (Figure 1) employs a reference database indexed to contain all sequences present in NCBI Nucleotide (nt) across all kingdoms of life (Martí *et al*. 2025) (database available here: https://benlangmead.github.io/aws-indexes/centrifuge). This enables broad, comprehensive taxonomic coverage of microbiome communities, including bacteria, fungi and host-associated eukaryotes, though at high computational cost. To provide a complementary resource for microbiome-centric model training, we leveraged high-performance computing (HPC) resources at Lawrence Livermore National Laboratory to perform metagenomic classification on 50TB of publicly available raw metagenomic sequence data. We generated structurally consistent microbiome taxonomy feature count tables for 13 897 samples, spanning across 84 studies (Karlsson *et al*. 2013, Loman *et al*. 2013, Qin *et al*. 2014, Zeller *et al*. 2014, Bäckhed *et al*. 2015, Bengtsson-Palme *et al*. 2015, Castro-Nallar *et al*. 2015, David *et al*. 2015, Feng *et al*. 2015, Sankaranarayanan *et al*. 2015, Shi *et al*. 2015, Chng *et al*. 2016, Heintz-Buschart *et al*. 2016, Vincent *et al*. 2016, Asnicar *et al*. 2017, 2021, Bedarf *et al*. 2017, Brooks *et al*. 2017, Byrd *et al*. 2017, Hall *et al*. 2017, Li *et al.* 2017, Jie *et al*. 2017, Loomba *et al*. 2017, Mancabelli *et al*. 2017, Nagy-Szakal *et al*. 2017, Oh *et al*. 2017, Olm *et al*. 2017, Piper *et al*. 2017, Wen *et al*. 2017, Yan *et al*. 2017, Yu *et al*. 2017, Zinkernagel *et al*. 2017, Deshpande *et al*. 2018, Espinoza *et al*. 2018, Gopalakrishnan *et al*. 2018, Hannigan *et al*. 2018, Kieser *et al*. 2018, Matson *et al*. 2018, McDonald *et al*. 2018, Rosa *et al*. 2018, Ye *et al*. 2018, Franzosa *et al*. 2019, Guillén *et al*. 2019, Gupta *et al*. 2019, Hu *et al*. 2019, Maya-Lucas *et al*. 2019, Proctor *et al*. 2019, Qi *et al*. 2019, Ventura *et al*. 2019, Weng *et al*. 2019, Yachida *et al*. 2019, Zhu *et al.* 2020, Ghensi *et al*. 2020, Polster *et al*. 2020, Pust *et al*. 2020, Qian *et al*. 2020, Rubel *et al*. 2020, Zuo *et al.* 2020, Zysset-Burri *et al*. 2020, Zhu *et al.* 2021, Tay *et al*. 2021, Bai *et al*. 2022, Bommana *et al*. 2022, Boolchandani *et al*. 2022, Chang *et al*. 2022, Jo *et al*. 2022, Laske *et al*. 2022, Lin *et al*. 2022, Liu *et al*. 2022, Nagata *et al*. 2022, Zuo *et al.* 2022, Wallen *et al*. 2022, Sun *et al.* 2022, Xiong *et al.* 2022, Zhang *et al*. 2022, Zhou *et al*. 2022, Boktor *et al*. 2023, Ferreiro *et al*. 2023, Guo *et al*. 2023, Sun *et al.* 2023, Khachatryan *et al*. 2023, Pienkowska *et al*. 2023, Xiong *et al.* 2023, Wang *et al.* 2023). We further performed extensive metadata curation of all assessed studies through a combination of manual curation and automated parsing, producing a consistent inter-study metadata table specifically structured to facilitate training of ML models for prediction of human health. We have since integrated this collection of microbiome and metadata datasets into machine learning models for disease state predictions (Goncalves *et al*. 2026, Zhu *et al*. 2025). In particular, we demonstrated that integrating microbiome profiles with metadata significantly improves classification across disease types with AUROC values reaching up to 0.757 and consistent improvements observed for cancer and neurological disease states. These curated feature tables, metadata and data descriptions are available at https://gdo-meta2db.llnl.gov/ and from Zenodo at https://zenodo.org/records/17315984.

While Meta2DB provides a harmonized resource for meta-analyses and machine learning applications, several limitations should be noted. Firstly, microbiome profiles were generated using the January 2023 version of the NCBI nt database at the time of analysis. Although microbial taxonomy and sequence repositories continue to evolve, benchmarking across subsequent nt releases using standardized mock communities showed minimal variation in precision and recall (Fig. S1, Table S1, available as supplementary data at *Bioinformatics* online). Effects may be more pronounced in complex or poorly characterized microbiomes, which is an important area for future work. In addition, although Meta2DB includes metadata describing technical factors and other covariates, the feature sets are not batch corrected. Users may apply methods such as ComBat, leveraging the provided metadata, and incorporate within-study controls and co-variates to adjust for study-specific biases. Such strategies are important for downstream prediction applications.

## 2 Methods

### 2.1 Selection of studies for meta-analysis

Literature studies were selected for data intake and curation if they met each of the following requirements: (1) biospecimens were processed via whole metagenome short-read sequencing using an Illumina platform, (2) raw sequence data and study metadata were publicly accessible, (3) the study was performed in the context of one or more defined human disease states, and (4) each assessed biospecimen could be assigned one of two binary labels corresponding to “control” or “diseased.” All included studies satisfy this binary labelling with the exception of Boolchandani *et al*. (2022), which included “pre-disease” samples representing baseline specimens collected prior to diarrhea onset.

The data collection process includes leveraging an existing resource of curated microbiome data and conducting a strategic search in PubMed. The curatedMetagenomicData package is a pre-existing, well-curated collection of studies, many of which met the characteristics described above (Pasolli *et al*. 2017). A subset of studies from this curated package was selected for inclusion. To supplement this list of studies, a literature search was carried out on PubMed to retrieve relevant articles. The following search terms were used in combination; “shotgun,” “metagenomics,” “disease,” and studies were manually curated to ensure that they met the requirements listed above.

These parameters were selected for consistency with the aim of facilitating downstream analyses; it is however acknowledged that future analyses could benefit from incorporation of additional data modalities such as amplicon sequences and long-read sequencing, which will be the subject of future iterations.

### 2.2 Metadata curation

Metadata was extracted from multiple potential sources depending on the individual study of interest, including from the publication and supplementary data or sequence repository. Data sources also included large databases, like the Inflammatory Bowel Disease database of the iHMP, which contains thousands of metagenomics samples from patients with either Crohn’s Disease (CD) or ulcerative colitis (UC), as well as control samples. The Genomic Standards Consortium’s Minimum Information about any Sequence (MIxS) metadata standards were used to group and standardize patient health, treatment, and demographic metadata across all studies. Controlled vocabularies for defined disorders and health states were used to create consistent formatting and naming. In addition to metadata common to all studies, metadata specific to each disorder was also preserved. Metadata around sample collection and type of assay performed were tracked to account for possible effects on outcomes. To distinguish different types of missing data, “not applicable” was assigned to fields irrelevant to a given sample, “not available” indicated relevant data that were missing, and “none” denoted cases where a treatment or observation was explicitly not administered or not observed in the host.

### 2.2 Raw sequence acquisition

Raw sequence data (FASTQ files) were downloaded from public repositories including the NCBI Sequence Read Archive (SRA) (Leinonen *et al*. 2011) and the European Nucleotide Archive (ENA) (Yuan *et al.* 2024). The Integrative Human Microbiome Project (iHMP) sequences were obtained from the HMP Data Coordination Center (Proctor *et al*. 2019).

### 2.3 Sequence pre-processing

Raw sequence data were pre-processed via fastp using default settings (Chen *et al*. 2018). These parameters include using a qualified quality phred value of Q15, an unqualified base percentage of 40%, and a minimum read length of 15. Individual post-filtering of samples was performed with the following read count thresholds: 5M paired-end reads for fecal samples and 1M paired-end reads for skin, oral, and nasal samples. Samples not meeting these thresholds were removed from downstream analysis. Reads were subsequently aligned to the GRCh38 human reference genome using minimap2 (Li 2018) for the removal of human reads.

### 2.4 Metagenomics classification

Resultant reads were processed for metagenomic classification via Centrifuge (Kim *et al*. 2016) against the NCBI Nucleotide (nt) database retrieved on 4 January 2023. This classification approach applies taxonomic labels via Lowest Common Ancestor (LCA) classification strategy. Database post-processing was previously described and includes quality control measures such as reference decontamination, masking of low complexity regions and filtering of short reads (<16nt) (Martí *et al*. 2025). A Minimum Hit Length (MHL) of 15 was employed for initial Centrifuge processing. Post-processing was performed via Recentrifuge (Marti 2019) to propagate and normalize Centrifuge assignment scores and perform additional filtering. For purposes of downstream analysis, taxonomic assignments were filtered at a MHL threshold of 40 and feature count tables were produced.

### 2.5 UMAP visualization

UMAP dimensionality reduction was applied to all 13 897 samples using the umap (version 0.2.10) package in R (version 4.2.2) with default parameters (n_neighbors = 15, n_components = 2, n_epochs = 200). The local neighborhood structure was constructed using the UMAP algorithm on pairwise Bray-Curtis distances.

## Supplementary Material

btag422_Supplementary_Data

## Data Availability

The processed feature tables, metadata and data descriptions are freely available for download in CSV format at https://gdo-meta2db.llnl.gov/ and at https://zenodo.org/records/17315984. The indexed version of the centrifuge database based on the entirety of NCBI nt is available at https://benlangmead.github.io/aws-indexes/centrifuge.

## References

[btag422-B1] Abdill RJ , Graham SP, Rubinetti V et al 2023. Integration of 168,000 samples reveals global patterns of the human gut microbiome. Cell 2025;188:1100–18. 10.1016/j.cell.2024.12.017 PMC11848717 39848248

[btag422-B2] Asnicar F , Berry SE, Valdes AM et al Microbiome connections with host metabolism and habitual diet from 1,098 deeply phenotyped individuals. Nat Med 2021;27:321–32. 10.1038/s41591-020-01183-8. 33432175 PMC8353542

[btag422-B3] Asnicar F , Manara S, Zolfo M et al Studying vertical microbiome transmission from mothers to infants by strain-level metagenomic profiling. mSystems 2017;2:e00164-16. 10.1128/mSystems.00164-16. 28144631 PMC5264247

[btag422-B4] Bäckhed F , Roswall J, Peng Y et al Dynamics and stabilization of the human gut microbiome during the first year of life. Cell Host Microbe 2015;17:690–703. 10.1016/j.chom.2015.04.004. 25974306

[btag422-B5] Bai X , Narayanan A, Skagerberg M et al Characterization of the upper respiratory bacterial microbiome in critically ill COVID-19 patients. Biomedicines 2022;10:982. https://www.mdpi.com/2227-9059/10/5/982. 35625719 10.3390/biomedicines10050982 PMC9138573

[btag422-B6] Bedarf JR , Hildebrand F, Coelho LP et al Functional implications of microbial and viral gut metagenome changes in early stage L-DOPA-naïve Parkinson’s disease patients. Genome Med 2017;9:39. 10.1186/s13073-017-0428-y. 28449715 PMC5408370

[btag422-B7] Beghini F , McIver LJ, Blanco-Míguez A et al Integrating taxonomic, functional, and strain-level profiling of diverse microbial communities with bioBakery 3. Elife 2021;10:e65088. 10.7554/eLife.65088. 33944776 PMC8096432

[btag422-B8] Bengtsson-Palme J , Angelin M, Huss M et al The human gut microbiome as a transporter of antibiotic resistance genes between continents. Antimicrob Agents Chemother 2015;59:6551–60. 10.1128/aac.00933-15. 26259788 PMC4576037

[btag422-B9] Boktor JC , Sharon G, Verhagen Metman LA et al Integrated multi-cohort analysis of the Parkinson’s disease gut metagenome. Mov Disord 2023;38:399–409. 10.1002/mds.29300. 36691982

[btag422-B10] Bommana S , Richards G, Kama M et al Metagenomic shotgun sequencing of endocervical, vaginal, and rectal samples among Fijian women with and without *Chlamydia trachomatis* reveals disparate microbial populations and function across anatomic sites: a pilot study. Microbiol Spectr 2022;10:e0010522. 10.1128/spectrum.00105-22. 35579443 PMC9241848

[btag422-B11] Boolchandani M , Blake KS, Tilley DH et al Impact of international travel and diarrhea on gut microbiome and resistome dynamics. Nat Commun 2022;13:7485. 10.1038/s41467-022-34862-w. 36470885 PMC9722912

[btag422-B12] Brooks B , Olm MR, Firek BA et al Strain-resolved analysis of hospital rooms and infants reveals overlap between the human and room microbiome. Nat Commun 2017;8:1814. 10.1038/s41467-017-02018-w. 29180750 PMC5703836

[btag422-B13] Byrd AL , Deming C, Cassidy SKB et al *Staphylococcus aureus* and *Staphylococcus epidermidis* strain diversity underlying pediatric atopic dermatitis. Sci Transl Med 2017;9:eaal4651. 10.1126/scitranslmed.aal4651. 28679656 PMC5706545

[btag422-B14] Callahan BJ , McMurdie PJ, Rosen MJ et al DADA2: high-resolution sample inference from illumina amplicon data. Nat Methods 2016;13:581–3. 10.1038/nmeth.3869. 27214047 PMC4927377

[btag422-B15] Castro-Nallar E , Bendall ML, Pérez-Losada M et al Composition, taxonomy and functional diversity of the oropharynx microbiome in individuals with schizophrenia and controls. PeerJ 2015;3:e1140. 10.7717/peerj.1140. 26336637 PMC4556144

[btag422-B16] Chang H-W , Yan D, Singh R et al Multiomic analysis of the gut microbiome in psoriasis reveals distinct host–microbe associations. JID Innovations 2022;2:100115. 10.1016/j.xjidi.2022.100115. 35757783 PMC9214347

[btag422-B17] Chen S , Zhou Y, Chen Y et al FASTP: an ultra-fast all-in-one FASTQ preprocessor. Bioinformatics 2018;34:i884–90. 10.1093/bioinformatics/bty560. 30423086 PMC6129281

[btag422-B18] Chng KR , Tay AS, Li C et al Whole metagenome profiling reveals skin microbiome-dependent susceptibility to atopic dermatitis flare. Nat Microbiol 2016;1:16106. 10.1038/nmicrobiol.2016.106. 27562258

[btag422-B19] David LA , Weil A, Ryan ET et al Gut microbial succession follows acute secretory diarrhea in humans. mBio 2015;6:e00381-15. 10.1128/mBio.00381-15. 25991682 PMC4442136

[btag422-B20] Deshpande NP , Riordan SM, Castaño-Rodríguez N et al Signatures within the esophageal microbiome are associated with host genetics, age, and disease. Microbiome 2018;6:227. 10.1186/s40168-018-0611-4. 30558669 PMC6297961

[btag422-B21] Espinoza JL , Harkins DM, Torralba M et al Supragingival plaque microbiome ecology and functional potential in the context of health and disease. mBio 2018;9:e01631-18. 10.1128/mBio.01631-18. 30482830 PMC6282201

[btag422-B22] Feng Q , Liang S, Jia H et al Gut microbiome development along the colorectal adenoma-carcinoma sequence. Nat Commun 2015;6:6528. 10.1038/ncomms7528. 25758642

[btag422-B23] Ferreiro AL , Choi J, Ryou J et al Gut microbiome composition may be an indicator of preclinical alzheimer’s disease. Sci Transl Med 2023;15:eabo2984. 10.1126/scitranslmed.abo2984 37315112 PMC10680783

[btag422-B24] Franzosa EA , Sirota-Madi A, Avila-Pacheco J et al Gut microbiome structure and metabolic activity in inflammatory bowel disease. Nat Microbiol 2019;4:293–305. 10.1038/s41564-018-0306-4. 30531976 PMC6342642

[btag422-B25] Fung DLX , Li X, Leung CK et al A self-knowledge distillation-driven CNN-LSTM model for predicting disease outcomes using longitudinal microbiome data. Bioinform Adv 2023;3:vbad059. 10.1093/bioadv/vbad059. 37228387 PMC10203376

[btag422-B26] Ghensi P , Manghi P, Zolfo M et al Strong oral plaque microbiome signatures for dental implant diseases identified by strain-resolution metagenomics. NPJ Biofilms Microbiomes 2020;6:47. 10.1038/s41522-020-00155-7. 33127901 PMC7603341

[btag422-B27] Goncalves AR , Ranganathan H, Valdes C et al Beyond microbial abundance: metadata integration enhances disease prediction in human microbiome studies. Front Microbiol 2026;16:1695501. 10.3389/fmicb.2025.1695501. PMC12869998 41648004

[btag422-B28] Gopalakrishnan V , Spencer CN, Nezi L et al Gut microbiome modulates response to anti-PD-1 immunotherapy in melanoma patients. Science 2018;359:97–103. 10.1126/science.aan4236. 29097493 PMC5827966

[btag422-B29] Guillén Y , Noguera-Julian M, Rivera J et al Low nadir CD4+ T-cell counts predict gut dysbiosis in HIV-1 infection. Mucosal Immunol 2019;12:232–46. 10.1038/s41385-018-0083-7. 30171206

[btag422-B30] Guo C , Che X, Briese T et al Deficient butyrate-producing capacity in the gut microbiome is associated with bacterial network disturbances and fatigue symptoms in ME/CFS. Cell Host Microbe 2023;31:288–304.e8. 10.1016/j.chom.2023.01.004. 36758522 PMC10183837

[btag422-B31] Gupta A , Dhakan DB, Maji A et al Association of *Flavonifractor plautii*, a flavonoid-degrading bacterium, with the gut microbiome of colorectal cancer patients in India. mSystems 2019;4:e00438–19. 10.1128/mSystems.00438-19. 31719139 PMC7407896

[btag422-B32] Hall AB , Yassour M, Sauk J et al A novel *Ruminococcus gnavus* clade enriched in inflammatory bowel disease patients. Genome Med 2017;9:103. 10.1186/s13073-017-0490-5. 29183332 PMC5704459

[btag422-B33] Hannigan GD , Duhaime MB, Ruffin MT et al Diagnostic potential and interactive dynamics of the colorectal cancer virome. mBio 2018;9:e02248–18. 10.1128/mBio.02248-18. 30459201 PMC6247079

[btag422-B34] Heintz-Buschart A , May P, Laczny CC et al Integrated multi-omics of the human gut microbiome in a case study of familial type 1 diabetes. Nat Microbiol 2016;2:16180. 10.1038/nmicrobiol.2016.180. 27723761

[btag422-B35] Hu Y , Feng Y, Wu J et al The gut microbiome signatures discriminate healthy from pulmonary tuberculosis patients. Front Cell Infect Microbiol 2019;9:90. 10.3389/fcimb.2019.00090. 31001490 PMC6456665

[btag422-B36] Jie Z , Xia H, Zhong SL et al The gut microbiome in atherosclerotic cardiovascular disease. Nat Commun 2017;8:845. 10.1038/s41467-017-00900-1. 29018189 PMC5635030

[btag422-B37] Jo S , Kang W, Hwang YS et al Oral and gut dysbiosis leads to functional alterations in Parkinson’s disease. NPJ Parkinsons Dis 2022;8:87. 10.1038/s41531-022-00351-6. 35798742 PMC9262988

[btag422-B38] Karlsson FH , Tremaroli V, Nookaew I et al Gut metagenome in European women with normal, impaired and diabetic glucose control. Nature 2013;498:99–103. 10.1038/nature12198. 23719380

[btag422-B39] Khachatryan L , Xiang Y, Ivanov A et al Results and lessons learned from the sbv IMPROVER metagenomics diagnostics for inflammatory bowel disease challenge. Sci Rep 2023;13:6303. 10.1038/s41598-023-33050-0. 37072468 PMC10113391

[btag422-B40] Kieser S , Sarker SA, Sakwinska O et al Bangladeshi children with acute diarrhoea show faecal microbiomes with increased Streptococcus abundance, irrespective of diarrhoea aetiology. Environ Microbiol 2018;20:2256–69. 10.1111/1462-2920.14274. 29786169

[btag422-B41] Kim D , Song L, Breitwieser FP et al Centrifuge: rapid and sensitive classification of metagenomic sequences. Genome Res 2016;26:1721–9. 10.1101/gr.210641.116. 27852649 PMC5131823

[btag422-B42] Kuhn M , Thomas SB, Schmidt P et al Metalog: curated and harmonised contextual data for global metagenomics samples. Nucleic Acids Res 2026;54:D826–34. 10.1093/nar/gkaf1118. 41171125 PMC12807751

[btag422-B43] Laske C , Müller S, Preische O et al Signature of Alzheimer’s disease in intestinal microbiome: results from the AlzBiom study. Front Neurosci 2022;16:792996. 10.3389/fnins.2022.792996. 35516807 PMC9063165

[btag422-B44] Leinonen R , Sugawara H, Shumway M. The sequence read archive. Nucleic Acids Res 2011;39:D19–21. 10.1093/nar/gkq1019. 21062823 PMC3013647

[btag422-B45] Li H. Minimap2: pairwise alignment for nucleotide sequences. Bioinformatics 2018;34:3094–100. 10.1093/bioinformatics/bty191. 29750242 PMC6137996

[btag422-B46] Li J , Zhao F, Wang Y et al Gut microbiota dysbiosis contributes to the development of hypertension. Microbiome 2017;5:14. 10.1186/s40168-016-0222-x. 28143587 PMC5286796

[btag422-B47] Lin B , Wang M, Gao R et al Characteristics of gut microbiota in patients with GH-Secreting pituitary adenoma. Microbiol Spectr 2022;10:e0042521. 10.1128/spectrum.00425-21. 35019688 PMC8754134

[btag422-B48] Liu Q , Mak JWY, Su Q et al Gut microbiota dynamics in a prospective cohort of patients with post-acute COVID-19 syndrome. Gut 2022;71:544–52. 10.1136/gutjnl-2021-325989. 35082169

[btag422-B49] Lo C , Marculescu R. MetaNN: accurate classification of host phenotypes from metagenomic data using neural networks. BMC Bioinformatics 2019;20:314. 10.1186/s12859-019-2833-2. 31216991 PMC6584521

[btag422-B50] Loman NJ , Constantinidou C, Christner M et al A culture-independent sequence-based metagenomics approach to the investigation of an outbreak of Shiga-toxigenic *Escherichia coli* O104: h 4. Jama 2013;309:1502–10. 10.1001/jama.2013.3231. 23571589

[btag422-B51] Loomba R , Seguritan V, Li W et al Gut microbiome-based metagenomic signature for non-invasive detection of advanced fibrosis in human nonalcoholic fatty liver disease. Cell Metab 2017;25:1054–62.e5. 10.1016/j.cmet.2017.04.001. 28467925 PMC5502730

[btag422-B52] Mancabelli L , Milani C, Lugli GA et al Unveiling the gut microbiota composition and functionality associated with constipation through metagenomic analyses. Sci Rep 2017;7:9879. 10.1038/s41598-017-10663-w. 28852182 PMC5575163

[btag422-B53] Marti JM. Recentrifuge: robust comparative analysis and contamination removal for metagenomics. PLoS Comput Biol 2019;15:e1006967. 10.1371/journal.pcbi.1006967. 30958827 PMC6472834

[btag422-B54] Martí JM , Kok CR, Thissen JB et al Addressing the dynamic nature of reference data: a new nucleotide database for robust metagenomic classification. mSystems 2025;10:e01239–24. doi:10.1128/msystems.01239-24. 40111052 10.1128/msystems.01239-24 PMC12013259

[btag422-B55] Matson V , Fessler J, Bao R et al The commensal microbiome is associated with anti-PD-1 efficacy in metastatic melanoma patients. Science 2018;359:104–8. 10.1126/science.aao3290. 29302014 PMC6707353

[btag422-B56] Maya-Lucas O , Murugesan S, Nirmalkar K et al The gut microbiome of Mexican children affected by obesity. Anaerobe 2019;55:11–23. 10.1016/j.anaerobe.2018.10.009. 30366118

[btag422-B57] McDonald D , Hyde E, Debelius JW et al American gut: an open platform for citizen science microbiome research. mSystems 2018;3:e00031–18. 10.1128/msystems.00031-18. 29795809 PMC5954204

[btag422-B58] Nagata N , Nishijima S, Kojima Y et al Metagenomic identification of microbial signatures predicting pancreatic cancer from a multinational study. Gastroenterology 2022;163:222–38. 10.1053/j.gastro.2022.03.054. 35398347

[btag422-B59] Nagy-Szakal D , Williams BL, Mishra N et al Fecal metagenomic profiles in subgroups of patients with myalgic encephalomyelitis/chronic fatigue syndrome. Microbiome 2017;5:44. 10.1186/s40168-017-0261-y. 28441964 PMC5405467

[btag422-B60] Namkung J. Machine learning methods for microbiome studies. J Microbiol 2020;58:206–16. 10.1007/s12275-020-0066-8. 32108316

[btag422-B61] Oh S , Yap GC, Hong PY et al Immune-modulatory genomic properties differentiate gut microbiota of infants with and without eczema. PLoS One 2017;12:e0184955. 10.1371/journal.pone.0184955. 29049378 PMC5648123

[btag422-B62] Olm MR , Brown CT, Brooks B et al Identical bacterial populations colonize premature infant gut, skin, and oral microbiomes and exhibit different in situ growth rates. Genome Res 2017;27:601–12. 10.1101/gr.213256.116. 28073918 PMC5378178

[btag422-B63] Pasolli E , Schiffer L, Manghi P et al Accessible, curated metagenomic data through ExperimentHub. Nat Methods 2017a;14:1023–4. 10.1038/nmeth.4468. 29088129 PMC5862039

[btag422-B65] Pienkowska K , Pust MM, Gessner M et al The cystic fibrosis upper and lower airway metagenome. Microbiol Spectr 2023;11:e0363322. 10.1128/spectrum.03633-22. 36892308 PMC10101124

[btag422-B66] Piper HG , Fan D, Coughlin LA et al Severe gut microbiota dysbiosis is associated with poor growth in patients with short bowel syndrome. JPEN J Parenter Enteral Nutr 2017;41:1202–12. 10.1177/0148607116658762. 27406942

[btag422-B67] Polster SP , Sharma A, Tanes C et al Permissive microbiome characterizes human subjects with a neurovascular disease cavernous angioma. Nat Commun 2020;11:2659. 10.1038/s41467-020-16436-w. 32461638 PMC7253448

[btag422-B68] Proctor LM , Heather H, Creasy JM et al The integrative human microbiome project. Nature 2019;569:641–8. 10.1038/s41586-019-1238-8. 31142853 PMC6784865

[btag422-B69] Pust MM , Wiehlmann L, Davenport C et al The human respiratory tract microbial community structures in healthy and cystic fibrosis infants. NPJ Biofilms Microbiomes 2020;6:61. 10.1038/s41522-020-00171-7. 33319812 PMC7738502

[btag422-B70] Qi X , Yun C, Sun L et al Gut microbiota-bile acid-interleukin-22 axis orchestrates polycystic ovary syndrome. Nat Med 2019;25:1225–33. 10.1038/s41591-019-0509-0. 31332392 PMC7376369

[btag422-B71] Qian Y , Yang X, Xu S et al Gut metagenomics-derived genes as potential biomarkers of Parkinson’s disease. Brain 2020;143:2474–89. 10.1093/brain/awaa201. 32844199

[btag422-B72] Qin N , Yang F, Li A et al Alterations of the human gut microbiome in liver cirrhosis. Nature 2014;513:59–64. 10.1038/nature13568. 25079328

[btag422-B73] Rosa BA , Supali T, Gankpala L et al Differential human gut microbiome assemblages during soil-transmitted helminth infections in Indonesia and Liberia. Microbiome 2018;6:33. 10.1186/s40168-018-0416-5. 29486796 PMC6389212

[btag422-B74] Rubel MA , Abbas A, Taylor LJ et al Lifestyle and the presence of helminths is associated with gut microbiome composition in cameroonians. Genome Biol 2020;21:122. 10.1186/s13059-020-02020-4. 32450885 PMC7249393

[btag422-B75] Sankaranarayanan K , Ozga AT, Warinner C et al Gut microbiome diversity among cheyenne and arapaho individuals from Western Oklahoma. Curr Biol 2015;25:3161–9. 10.1016/j.cub.2015.10.060. 26671671 PMC4703035

[btag422-B76] Sharma D , Paterson AD, Xu W. TaxoNN: ensemble of neural networks on stratified microbiome data for disease prediction. Bioinformatics 2020;36:4544–50. 10.1093/bioinformatics/btaa542. 32449747 PMC7750934

[btag422-B77] Shi B , Chang M, Martin J et al Dynamic changes in the subgingival microbiome and their potential for diagnosis and prognosis of periodontitis. mBio 2015;6:e01926–14. 10.1128/mBio.01926-14. 25691586 PMC4337560

[btag422-B78] Sun H , Guo Y, Wang H et al Gut commensal *Parabacteroides distasonis* alleviates inflammatory arthritis. Gut 2023;72:1664–77. 10.1136/gutjnl-2022-327756. 36604114

[btag422-B79] Sun Z , Zhang M, Li M et al Interactions between human gut microbiome dynamics and Sub-Optimal health symptoms during seafaring expeditions. Microbiol Spectr 2022;10:e0092521. 10.1128/spectrum.00925-21. 35019672 PMC8754112

[btag422-B80] Tay ASL , Li C, Nandi T et al Atopic dermatitis microbiomes stratify into ecologic dermotypes enabling microbial virulence and disease severity. J Allergy Clin Immunol 2021;147:1329–40. 10.1016/j.jaci.2020.09.031. 33039480

[btag422-B81] Ventura RE , Iizumi T, Battaglia T et al Gut microbiome of treatment-naïve MS patients of different ethnicities early in disease course. Sci Rep 2019;9:16396. 10.1038/s41598-019-52894-z. 31705027 PMC6841666

[btag422-B82] Vincent C , Miller MA, Edens TJ et al Bloom and bust: intestinal microbiota dynamics in response to hospital exposures and *Clostridium difficile* colonization or infection. Microbiome 2016;4:12. 10.1186/s40168-016-0156-3. 26975510 PMC4791782

[btag422-B83] Wallen ZD , Demirkan A, Twa G et al Metagenomics of Parkinson’s disease implicates the gut microbiome in multiple disease mechanisms. Nat Commun 2022;13:6958. 10.1038/s41467-022-34667-x. 36376318 PMC9663292

[btag422-B84] Wang Y , Bhattacharya T, Jiang Y et al A novel deep learning method for predictive modeling of microbiome data. Brief Bioinform 2021;22:bbaa073. 10.1093/bib/bbaa073. 32406914 PMC13017428

[btag422-B85] Wang Z , Liang L, Liu L et al Changes in the gut microbiome associated with intussusception in patients with Peutz-Jeghers syndrome. Microbiol Spectr 2023;11:e0281922. 10.1128/spectrum.02819-22. 36719190 PMC10101062

[btag422-B86] Wen C , Zheng Z, Shao T et al Quantitative metagenomics reveals unique gut microbiome biomarkers in ankylosing spondylitis. Genome Biol 2017;18:142. 10.1186/s13059-017-1271-6. 28750650 PMC5530561

[btag422-B87] Weng YJ , Gan HY, Li X et al Correlation of diet, microbiota and metabolite networks in inflammatory bowel disease. J Dig Dis 2019;20:447–59. 10.1111/1751-2980.12795. 31240835

[btag422-B88] Xiong R , Gunter C, Fleming E et al Multi-'omics of gut microbiome-host interactions in short- and long-term myalgic encephalomyelitis/chronic fatigue syndrome patients. Cell Host Microbe 2023;31:273–87.e5. 10.1016/j.chom.2023.01.001. 36758521 PMC10353054

[btag422-B89] Xiong Z , Peng K, Song S et al Cerebral intraparenchymal hemorrhage changes patients’ gut bacteria composition and function. Front Cell Infect Microbiol 2022;12:829491. 10.3389/fcimb.2022.829491. 35372117 PMC8966894

[btag422-B90] Yachida S , Mizutani S, Shiroma H et al Metagenomic and metabolomic analyses reveal distinct stage-specific phenotypes of the gut microbiota in colorectal cancer. Nat Med 2019;25:968–76. 10.1038/s41591-019-0458-7. 31171880

[btag422-B91] Yan Q , Gu Y, Li X et al Alterations of the gut microbiome in hypertension. Front Cell Infect Microbiol 2017;7:381. 10.3389/fcimb.2017.00381. 28884091 PMC5573791

[btag422-B92] Ye Z , Zhang N, Wu C et al A metagenomic study of the gut microbiome in Behcet’s disease. Microbiome 2018;6:135. 10.1186/s40168-018-0520-6. 30077182 PMC6091101

[btag422-B93] Yu J , Feng Q, Wong SH et al Metagenomic analysis of faecal microbiome as a tool towards targeted non-invasive biomarkers for colorectal cancer. Gut 2017;66:70–8. 10.1136/gutjnl-2015-309800. 26408641

[btag422-B94] Yuan D , Ahamed A, Burgin J et al The European Nucleotide Archive in 2023. Nucleic Acids Res 2024;52:D92–7. 10.1093/nar/gkad1067. 37956313 PMC10767888

[btag422-B95] Zeller G , Tap J, Voigt AY et al Potential of fecal microbiota for early-stage detection of colorectal cancer. Mol Syst Biol 2014;10:766. 10.15252/msb.20145645. 25432777 PMC4299606

[btag422-B96] Zhai Z , Che X, Shen W et al HLRMDB: a comprehensive database of the human microbiome with metagenomic assembly, taxonomic classification, and functional annotation by analysis of long-read and hybrid sequencing data. Nucleic Acids Res 2026;54:D763–75. 10.1093/nar/gkaf1152. 41207298 PMC12807619

[btag422-B97] Zhang F , Wan Y, Zuo T et al Prolonged impairment of short-chain fatty acid and L-Isoleucine biosynthesis in gut microbiome in patients with COVID-19. Gastroenterology 2022;162:548–61.e4. 10.1053/j.gastro.2021.10.013. 34687739 PMC8529231

[btag422-B98] Zhou T , Wu J, Zeng Y et al SARS-CoV-2 triggered oxidative stress and abnormal energy metabolism in gut microbiota. MedComm (2020) 2022;3:e112. 10.1002/mco2.112. 35281785 PMC8906553

[btag422-B99] Zhu F , Ju Y, Wang W et al Metagenome-wide association of gut microbiome features for schizophrenia. Nat Commun 2020;11:1612. 10.1038/s41467-020-15457-9. 32235826 PMC7109134

[btag422-B100] Zhu H , Goncalves AR, Valdes C et al Hierarchical sparse Bayesian multitask model with scalable inference for microbiome analysis. arXiv preprint arXiv : 2502.02552, 2025, preprint: not peer reviewed.

[btag422-B101] Zhu Q , Hou Q, Huang S et al Compositional and genetic alterations in Graves’ disease gut microbiome reveal specific diagnostic biomarkers. ISME J 2021;15:3399–411. 10.1038/s41396-021-01016-7. 34079079 PMC8528855

[btag422-B102] Zinkernagel MS , Zysset-Burri DC, Keller I et al Association of the intestinal microbiome with the development of neovascular age-related macular degeneration. Sci Rep 2017;7:40826. 10.1038/srep40826. 28094305 PMC5240106

[btag422-B103] Zuo T , Zhang F, Lui GCY et al Alterations in gut microbiota of patients with COVID-19 during time of hospitalization. Gastroenterology 2020;159:944–55.e8. 10.1053/j.gastro.2020.05.048. 32442562 PMC7237927

[btag422-B104] Zuo W , Wang B, Bai X et al 16S rRNA and metagenomic shotgun sequencing data revealed consistent patterns of gut microbiome signature in pediatric ulcerative colitis. Sci Rep 2022;12:6421. 10.1038/s41598-022-07995-7. 35440670 PMC9018687

[btag422-B105] Zysset-Burri DC , Keller I, Berger LE et al Associations of the intestinal microbiome with the complement system in neovascular age-related macular degeneration. NPJ Genom Med 2020;5:34. 10.1038/s41525-020-00141-0. 32922859 PMC7463023

