# Supplementary material for: Meta2DB: curated shotgun metagenomic feature sets and metadata for health state prediction
Source: Bioinformatics. 2026 Jul 1;42(7):btag422. doi: 10.1093/bioinformatics/btag422 (PMC13335476; doi:10.1093/bioinformatics/btag422)
Supplement: btag422_Supplementary_Data [file btag422_supplementary_data.docx]

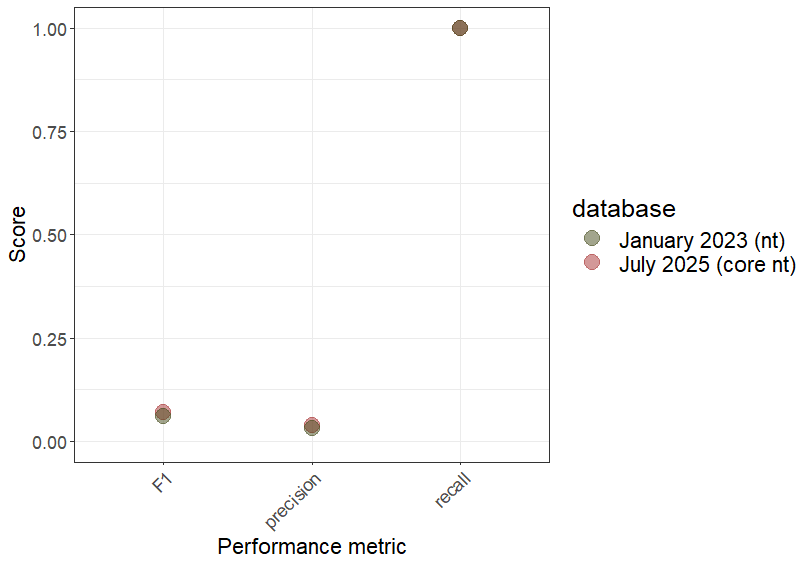


Fig S1. F1 score, precision and recall benchmarked across 12 standardized DNA mock communities. Standard controls were processed using Centrifuge against the January 2023 nt release used in Meta2DB and a more recent July 2025 core nt release. These results show minimal impact of the assessed database versions on taxonomic classification performance.

Table S1. Species composition and relative abundances (%) of standardized genomic DNA mock communities used for database benchmarking. Each sample represents a defined mixture of bacterial, fungal or eukaryotic organism spanning a range of taxonomic groups and abundance distributions.

| **sample** | **organism** | **abundance** |
| --- | --- | --- |
| zymo gut microbiome | *Faecalibacterium prausnitzii* | 14 |
|  | *Veillonella rogosae* | 14 |
|  | *Roseburia hominis* | 14 |
|  | *Bacteroides fragilis* | 14 |
|  | *Prevotella corporis* | 6 |
|  | *Bifidobacterium adolescentis* | 6 |
|  | *Fusobacterium nucleatum* | 6 |
|  | *Limosilactobacillus fermentum* | 6 |
|  | *Clostridioides difficile* | 1.5 |
|  | *Akkermansia muciniphila* | 1.5 |
|  | *Methanobrevibacter smithii* | 0.1 |
|  | *Salmonella enterica* | 0.01 |
|  | *Enterococcus faecalis* | 0.001 |
|  | *Clostridium perfringens* | 0.0001 |
|  | *Escherichia coli* | 14 |
|  | *Candida albicans* | 1.5 |
|  | *Saccharomyces cerevisiae* | 1.4 |
| zymo micro comm II | *Listeria monocytogenes* | 89.1 |
|  | *Pseudomonas aeruginosa* | 8.9 |
|  | *Bacillus subtilis* | 0.89 |
|  | *Saccharomyces cerevisiae* | 0.89 |
|  | *Escherichia coli* | 0.089 |
|  | *Salmonella enterica* | 0.089 |
|  | *Limosilactobacillus fermentum* | 0.0089 |
|  | *Enterococcus faecalis* | 0.00089 |
|  | *Cryptococcus neoformans* | 0.00089 |
|  | *Staphylococcus aureus* | 0.000089 |
| atcc mycobiome | *Aspergillus fumigatus* | 10 |
|  | *Cryptococcus neoformans* | 10 |
|  | *Trichophyton interdigitale* | 10 |
|  | *Penicillium chrysogenum* | 10 |
|  | *Fusarium keratoplasticum* | 10 |
|  | *Candida albicans* | 10 |
|  | *Nakaseomyces glabratus* | 10 |
|  | *Malassezia globosa* | 10 |
|  | *Saccharomyces cerevisiae* | 10 |
|  | *Cutaneotrichosporon dermatis* | 10 |
| atcc oral spike | *Homo sapiens* | 36 |
|  | *Schaalia odontolytica* | 10.7 |
|  | *Prevotella melaninogenica* | 10.7 |
|  | *Fusobacterium nucleatum* | 10.7 |
|  | *Streptococcus mitis* | 10.7 |
|  | *Veillonella parvula* | 10.7 |
|  | *Haemophilus parainfluenzae* | 10.7 |
| atcc skin spike | *Homo sapiens* | 28 |
|  | *Acinetobacter johnsonii* | 12 |
|  | *Corynebacterium striatum* | 12 |
|  | *Micrococcus luteus* | 12 |
|  | *Cutibacterium acnes* | 12 |
|  | *Staphylococcus epidermidis* | 12 |
|  | *Streptococcus mitis* | 12 |
| atcc gut spike | *Homo sapiens* | 80 |
|  | *Bacteroides fragilis* | 1.6 |
|  | *Phocaeicola vulgatus* | 1.6 |
|  | *Bifidobacterium adolescentis* | 1.6 |
|  | *Clostridioides difficile* | 1.6 |
|  | *Enterococcus faecalis* | 1.6 |
|  | *Lactiplantibacillus plantarum* | 1.6 |
|  | *Enterobacter cloacae* | 1.6 |
|  | *Escherichia coli* | 1.6 |
|  | *Helicobacter pylori* | 1.6 |
|  | *Salmonella enterica* | 1.6 |
|  | *Yersinia enterocolitica* | 1.6 |
|  | *Fusobacterium nucleatum* | 1.6 |
| protozoa mix | *Acanthamoeba castellanii* | 50 |
|  | *Naegleria fowleri* | 50 |
| mammal equal mix | *Felis catus* | 9 |
|  | *Canis lupus* | 9 |
|  | *Bos taurus* | 9 |
|  | *Equus caballus* | 9 |
|  | *Homo sapiens* | 9 |
|  | *Macaca mulatta* | 9 |
|  | *Mus musculus* | 9 |
|  | *Rattus norvegicus* | 9 |
|  | *Sus scrofa* | 9 |
|  | *Oryctolagus cuniculus* | 9 |
|  | *Ovis aries* | 9 |
| non-mammal mix | *Alligator mississippiensis* | 10 |
|  | *Gallus gallus* | 10 |
|  | *Ictalurus punctatus* | 10 |
|  | *Arabidopsis thaliana* | 10 |
|  | *Gossypium hirsutum* | 10 |
|  | *Zea mays* | 10 |
|  | *Drosophila melanogaster* | 10 |
|  | *Ctenocephalides felis* | 10 |
|  | *Aedes aegypti* | 10 |
|  | *Rhipicephalus sanguineus* | 10 |
| mammal log mix | *Mus musculus* | 90 |
|  | *Oryctolagus cuniculus* | 9 |
|  | *Homo sapiens* | 0.9 |
|  | *Rattus norvegicus* | 0.09 |
|  | *Sus scrofa* | 0.009 |
|  | *Macaca mulatta* | 0.0009 |
|  | *Canis lupus* | 0.00009 |
| ATCC 20 strain | *Acinetobacter baumannii* | 0.18 |
|  | *Bacillus pacificus* | 1.8 |
|  | *Phocaeicola vulgatus* | 0.02 |
|  | *Bifidobacterium adolescentis* | 0.02 |
|  | *Clostridium beijerinckii* | 1.8 |
|  | *Cutibacterium acnes* | 0.18 |
|  | *Deinococcus radiodurans* | 0.02 |
|  | *Enterococcus faecalis* | 0.02 |
|  | *Escherichia coli* | 18 |
|  | *Helicobacter pylori* | 0.18 |
|  | *Lactobacillus gasseri* | 0.18 |
|  | *Neisseria meningitidis* | 0.18 |
|  | *Porphyromonas gingivalis* | 18 |
|  | *Pseudomonas aeruginosa* | 1.8 |
|  | *Cereibacter sphaeroides* | 18 |
|  | *Schaalia odontolytica* | 0.02 |
|  | *Staphylococcus aureus* | 1.8 |
|  | *Staphylococcus epidermidis* | 18 |
|  | *Streptococcus agalactiae* | 1.8 |
|  | *Streptococcus mutans* | 18 |
| ATCC path mix | *Acinetobacter baumannii* | 0.1 |
|  | *Enterococcus faecalis* | 0.7 |
|  | *Escherichia coli* | 1.4 |
|  | *Klebsiella pneumoniae* | 14.4 |
|  | *Neisseria meningitidis* | 28.9 |
|  | *Pseudomonas aeruginosa* | 0.3 |
|  | *Staphylococcus aureus* | 15.1 |
|  | *Streptococcus agalactiae* | 2.9 |
|  | *Streptococcus pneumoniae* | 28.9 |
|  | *Streptococcus pyogenes* | 7.2 |
